# CoVITEST: A Fast and Reliable Method to Monitor Anti-SARS-CoV-2 Specific T Cells From Whole Blood

**DOI:** 10.3389/fimmu.2022.848586

**Published:** 2022-07-05

**Authors:** Natalia Egri, Victoria Olivé, José Hernández-Rodríguez, Pedro Castro, Catherine De Guzman, Libertad Heredia, Ana Castellet Segura, M. Dolores Fernandez, Noemi de Moner, María Torradeflot, Judit Ballús, Robert Martinez, Mario Vazquez, Marta Vidal Costa, Carlota Dobaño, Massimiliano Mazza, Lucia Mazzotti, Mariona Pascal, Manel Juan, Europa Azucena González-Navarro, Hugo Calderón

**Affiliations:** ^1^ Department of Immunology, Centre de Diagnòstic Biomèdic, Hospital Clínic of Barcelona, Barcelona, Spain; ^2^ Institut d’Investigacions Biomèdiques August Pi i Sunyer (IDIBAPS), Barcelona, Spain; ^3^ Occupational Health Department, Hospital Clínic de Barcelona, Barcelona, Spain; ^4^ Universitat de Barcelona, Barcelona, Spain; ^5^ Vasculitis Research Unit and Autoinflammatory Diseases Clinical Unit, Department of Autoimmune Diseases, Hospital Clínic of Barcelona, Institut d’Investigacions Biomèdiques August Pi i Sunyer (IDIBAPS), University of Barcelona, Barcelona, Spain; ^6^ Medical Intensive Care Unit, Hospital Clinic de Barcelona, Barcelona, Spain; ^7^ ISGlobal, Hospital Clínic - Universitat de Barcelona, Barcelona, Spain; ^8^ CIBER Infectious Diseases (CIBERINFEC), Barcelona, Spain; ^9^ IRCCS Istituto Romagnolo per lo Studio dei Tumori (IRST) “Dino Amadori”, Meldola, FC, Italy; ^10^ Allergy Network ARADyAL, Instituto de Salud Carlos III, Madrid, Spain

**Keywords:** T cells, SARS-CoV-2, blood test, cellular immune response, COVID-19

## Abstract

Cellular and humoral immune responses are essential for COVID-19 recovery and protection against SARS-CoV-2 reinfection. To date, the evaluation of SARS-CoV-2 immune protection has mainly focused on antibody detection, generally disregarding the cellular response, or placing it in a secondary position. This phenomenon may be explained by the complex nature of the assays needed to analyze cellular immunity compared with the technically simple and automated detection of antibodies. Nevertheless, a large body of evidence supports the relevance of the T cell’s role in protection against SARS-CoV-2, especially in vulnerable individuals with a weakened immune system (such as the population over 65 and patients with immunodeficiencies). Here we propose to use CoVITEST (Covid19 anti-Viral Immunity based on T cells for Evaluation in a Simple Test), a fast, affordable and accessible in-house assay that, together with a diagnostic matrix, allows us to determine those patients who might be protected with SARS-CoV-2-reactive T cells. The method was established using healthy SARS-CoV-2-naïve donors pre- and post-vaccination (*n=*30), and further validated with convalescent COVID-19 donors (*n=*51) in a side-by-side comparison with the gold standard IFN-γ ELISpot. We demonstrated that our CoVITEST presented reliable and comparable results to those obtained with the ELISpot technique in a considerably shorter time (less than 8 hours). In conclusion, we present a simple but reliable assay to determine cellular immunity against SARS-CoV-2 that can be used routinely during this pandemic to monitor the immune status in vulnerable patients and thereby adjust their therapeutic approaches. This method might indeed help to optimize and improve decision-making protocols for re-vaccination against SARS-CoV-2, at least for some population subsets.

## Introduction

Severe acute respiratory syndrome coronavirus 2 (SARS-CoV-2), the virus that causes COVID-19 (CoronaVIrus Disease 2019) has infected millions of people around the world. COVID-19 can manifest with a wide spectrum of disease severity ranging from asymptomatic/mild forms to life-threatening pneumonia with multiple organ failure and death (1). The progress from the early stages of the pandemic to this day has been enormous thanks to natural immunization, vaccination programs and the better understanding of the disease Yet, due the emergence of new variants of concern and the presence of non-immunized people, the waning of immunity and immunocompromised patients (2), this pandemic still has a profound impact on society; from healthcare to gender equality and economy (3).

The relevance of the innate immune response against SARS-CoV-2 is unquestionable, however, both the humoral and cellular arms of adaptive immunity are essential for recovery and protection against SARS-CoV-2 reinfection (4, 5). Antibodies opsonizes free viral particles, promoting both virus elimination and neutralization of the interaction of the virus with its cellular receptors, such as Angiotensin Converting Enzyme 2 (ACE2) and Neuropilin-1. Neutralizing antibodies prevent virus entry into new cells, avoiding the spread of free viral particles (6). However, antibodies alone are unable to clear the virus once it is replicating inside the cells, and clearance of the established infection relies exclusively on T lymphocytes. T cells are also important to support B cell functions and antibody affinity maturation (7). In addition, apart from developing their own function, CD4+ T cells also assist the cytotoxic activity of CD8+ T cells. Furthermore, CD8+ T lymphocytes have full capacity to recognize and kill virus-infected cells, hence being the effector component that eliminates intracellular SARS-CoV-2 in COVID-19 patients.

Overall, it is well known that training of the adaptive immunity by SARS-CoV-2 infection and vaccines is a key component for the resolution of this pandemic (8). In this regard, several studies have demonstrated that the convalescent and vaccinated population develop both humoral and cellular immunity, which significantly protects them from severe disease and death (9). Unfortunately, some immunized individuals are known to lose this adaptive protection against the virus due to fading immune response related to advanced age, debilitating chronic diseases, pharmacological immunosuppression or other primary or secondary causes of immunodeficiency (2, 10). Although less understood, a small proportion of healthy individuals, especially those with less severe disease forms, never achieve seroconversion while they preserve cellular immunity that protects them from the virus (11, 12).

To date, the evaluation of the immune protection against SARS-CoV-2 has mainly focused on the detection of antibodies, generally disregarding the cellular response or relegating it to a secondary plane. This phenomenon is probably explained by the complex nature of the assays needed to analyze cellular immunity, as opposed to the technically simple and automated detection of antibodies. Assays for detecting T cell-specific responses are long, complex and labor-intensive methods: for instance, the gold-standard ELISpot is lengthy and technically demanding, requiring previous isolation of Peripheral Blood Mononuclear Cell (PBMCs).

We believe that knowing the adaptive immunological status against SARS-CoV-2, especially in the vulnerable population, is "extremely relevant to implement measures to protect those patients with negative cellular immunization. For that reason, herein we describe CoVITEST (Covid19 anti-Viral Immunity based on T cells for Evaluation in a Simple Test), a point-of-care, fast and scalable in-house assay to detect SARS-CoV-2-reactive T cells from blood, based on the detection of cytokine and T cell activation markers. This assay does not only offer relatively rapid results without the need for lymphocyte isolation from blood, but it is also reliable in comparison to other standard methods and allows distinguishing between CD4+ and CD8+ T cell responses. More importantly, with our matrix we have a method to determine the immunization status of patients of interest.

## Methods

### Human Subjects

All participants gave written informed consent, and eligible subjects were treated according to the Helsinki Declaration. Approval was obtained from the Ethics Committee of the Hospital Clinic of Barcelona (HCB/2020/0967). The cohorts groups were:

#### (a) Healthy SARS-CoV-2 Naïve Donors

Samples from 30 healthy adult donors were collected at the Hospital Clinic of Barcelona between October 2020 and April 2021. Subjects were identified as SARS-CoV-2 naïve through self-reporting as well as being double negative for serology and cellular response against the virus (ELISpot). The selected subjects were monitored for immunization after vaccination with either Pfizer**^®^** (BNT162b2™) or Moderna**^®^** (mRNA-1273™) mRNA vaccines, or ChAdOx1n Cov-19™ (Oxford-Astrazeneca**^®^**).

Blood samples were collected at two time-points: pre-vaccine baseline to exclude virus exposure (time-point 1) and two weeks post-second dose vaccination (time-point 2). All participants were otherwise healthy and did not report any history of chronic health conditions. The median age of this control group was 45 years (ranging from 22 to 66).

#### (b) Convalescent COVID-19 Donors

Fifty-one convalescent individuals with previous asymptomatic (n=2) or pauci-symptomatic/mild (n=49) COVID-19 were enrolled in the study. Study inclusion criteria were adult subjects (≥18 years) with a positive real-time reverse transcriptase PCR (RT-PCR) test for SARS-CoV-2 from a nasopharyngeal swabs at diagnosis. Blood samples were collected between two weeks and six months after the first positive RT-PCR test. Median age of the participants was 32.5 years (range 23-62). All were otherwise healthy at the time of the study and did not report any history of chronic health conditions.

### Sample Collection

**Figure 1:**
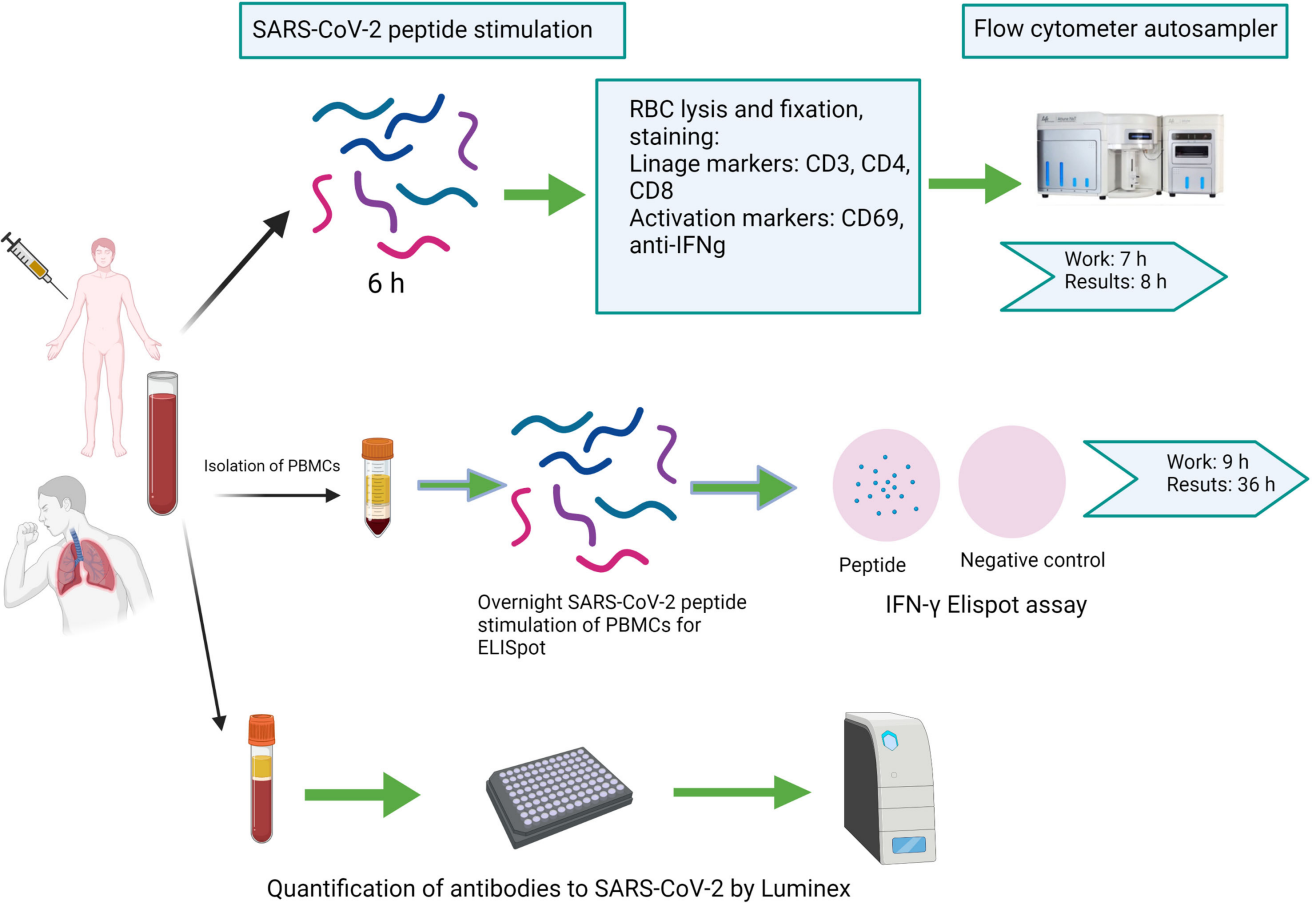
Schematic representation of the workflow for direct peptide stimulation of whole peripheral blood by CoVITEST, IFN-γELISpot or antibody quantification by Luminex^®^ (Created withBioRender.com).

Peripheral blood was collected from a forearm vein *via* a butterfly needle coupled to heparinized and serum tubes. To compare CoVITEST with the gold standard IFN-γELISpot assay, PBMCs were isolated from all collected blood samples by Ficoll-Paque density gradient centrifugation. For each subject, we analyzed samples according to the workflow described in **Figure 1**.

### CoVITEST (Assay for SARS-CoV-2-Specific T Cell Evaluation From Fresh Whole Blood)

Two hundred and fifty microliters of heparinized blood from each donor were aliquoted into three sterile vented-cap flow cytometry tubes. One tube was stimulated with a 1:1 mix of SARS-CoV-2 Spike and Nucleocapsid peptide pools (Miltenyi Biotec**^®^**) each at 2.5 µg/ml final concentration. Another tube was spiked with the equivalent volume of the peptide pool vehicle as the negative control and the third tube was stimulated with staphylococcal enterotoxin B superantigen (at 1 µg/ml final concentration) as the positive control. Tubes were then spiked with Fastimmune™ CD28/CD49d cocktail (BD Bioscience**^®^**) at 1 µg/ml final concentration, briefly vortexed at low speed before incubating at 37°C, 5% CO_2_ and 95% humidity. After 2 h of incubation, Brefeldin A was added at 1 µg/ml working concentration with a multi-dispenser pipette into all three tubes briefly shaking at low speed on a vortex before returning the samples to the incubator. Four hours later, samples were treated with EDTA (Invitrogen) at 2 mM final concentration, before a vigorous vortex and 15 min incubation at room temperature (RT).

The samples were then red-blood cell lysed and cells fixed with 2.5 mL of 1X BD FACS Lysing Solution (BD Bioscience**^®^**), gently mixed and incubated for 10 min at RT. Samples were centrifuged at 500 g for 5 min, and then the supernatant was decanted. Non-lysed cells were resuspended in 1.25 mL of 1X BD FACS Permeabilizing Solution 2 (BD Bioscience**^®^**) and incubated for 10 min at RT. Samples were further diluted with 3.5 mL of wash buffer and centrifuged at 500 g for 5 min, before decanting the supernatant.

Cell labelling was performed with fixation-compatible antibodies IFN-γ-FITC (Pharmingen**^®^**), CD69-PE, CD8-AF700, CD3-BV510 (BDBioscience**^®^**) and CD4-PerCP (Biolegend**^®^**) incubated with the permeabilized cells for 30 min at RT in dark condition. After the staining, cells were washed in FACS buffer, centrifuged at 500 g for 5 min; supernatants were then decanted before resuspending the cells in 1 ml of FACS buffer. All three tubes were stored in the dark at 4°C until acquisition (500 µl/tube) using an NxT Attune™ Flow Cytometer (ThermoFisher**^®^**)

**Figure 2:**
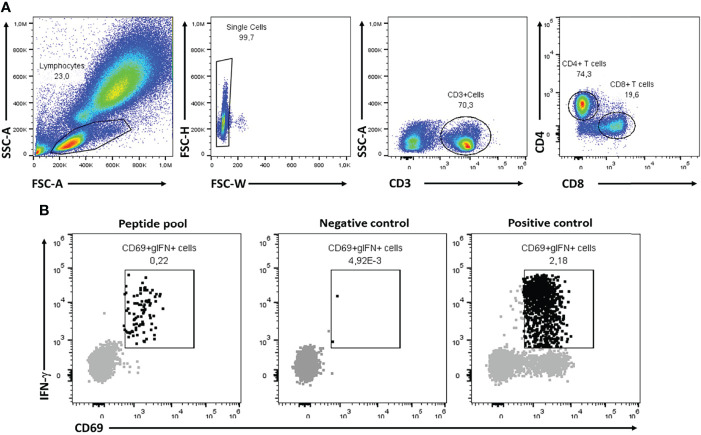
Representative flow cytometry analysis for CoVITEST. The flow cytometry gating strategy was performed sequentially; lymphocyte complexity (SSC *vs.* FSC), single cell, CD3+ and finally either CD4+ or CD8+ **(A)**. From there, 40,000 CD4+ or CD8+ T cells were further evaluated for CD69 and IFN- γ expression in S and N peptide pool, negative control (vehicle) and positive control (SEB) samples to determine the T cell reactivity for each donor **(B)**.

The samples were analyzed with Attune™ NxT Software, in accordance with the gating strategy shown in **Figure 2**. All four criteria stated below had to be met to determine a patient or donor as having cellular immunity against SARS-CoV-2:

- Fifteen or more events were detected from 40,000 CD4+ T cells in the test tube.
- IFN-γ MFI on CD4+IFN-γ+CD69+ events were 5000 or above.
- The number of events in the test tube was at least two-fold greater than in the unstimulated control tube.
- The positive control had more than 100 events.

The entire stimulation, staining and flow cytometry acquisition procedure can be performed in a sterile 96 deep-well plate instead of vented-cap cytometry tubes when testing multiple donors simultaneously (use a flow cytometer fitted with autosampler). When assessing multiple donors, peptide pools and controls can be prepared in a master mix with the Fastimmune cocktail and aliquoted *via* a multi-dispenser pipette.

### IFN-γELISpot

Stimulation was conducted with 2 × 10^5^ PBMCs in X-VIVO™ 15 medium (Lonza^®^) supplemented with 10% heat inactivated AB serum and PepTivator^®^ SARS-CoV-2 Prot_S and N peptide pools μg/ml, Miltenyi Biotec**^®^**). The diluent was PBS+DMSO with a final DMSO concentration of 1%. In the ELISpot negative control, X-VIVO 15 medium was employed with DMSO 20% to a final concentration of 1%. Negative control wells lacked peptides, and positive control wells included mAb CD3-2 from the kit. Cells were incubated overnight (16–20 h) at 37°C 5% CO2 in precoated anti-IFN-γ MSIP white plates (Human IFNg ELISpotPRO kit (ALP) (Mabtech) Ref: 3420-2AST-2, Mabtech^®^).

Plates were then washed five-times with PBS (Sigma-Aldrich) and incubated for 2 h at RT with horseradish peroxidase (HRP)-conjugated anti-IFN-γ detection antibody (1 μg/ml; clone mAb-7B6-1; Mabtech**^®^**). After five further washes with PBS, tetramethylbenzidine (TMB) substrate was added and spots were counted using an automated ELISpot Reader System (Autoimmun Diagnostika GmbH**^®^**).

To quantify positive peptide-specific responses, spots in the unstimulated wells were subtracted from the peptide-stimulated wells and the results expressed as SFU (Spot Forming Units)/2x10^5^ PBMCs.

We determined SARS-CoV-2-specific spots by spot increment, defined as stimulated spot numbers ≥6 SFU/2 × 10^5^ PBMCs. This cutoff was defined calculating the mean ± 2 standard deviations in a group of healthy donors obtained prior to the start of the SARS-CoV-2 pandemic. Spot counting was carried out automatically and was manually re-evaluated in all cases.

### Quantification of Antibodies Against SARS-CoV-2 by Luminex^®^

To establish seroprevalence, we used a serological assay based on the Luminex**^®^** technique that has the benefit of a higher dynamic range compared to other assays, favoring the quantification of immunoglobulin levels. We measured antibodies against the Receptor-Binding Domain (RBD) of the spike glycoprotein of SARS-CoV-2 (13). Median fluorescent intensities (MFI) were exported using the xPONENT™ software. Assay cutoff was calculated as the mean plus 2 standard deviations of log10-transformed MFIs of a donor pool of 30 negative samples obtained before the COVID-19 pandemic. The data used for the calculations were the ratio of the MFI of the particular individual with the MFI obtained from the donor pool, and a value ≥1 was considered positive. Assay sensitivity and specificity was calculated using samples from participants previously diagnosed with COVID-19 and more than 10 days after the onset of symptoms, being 97% for IgG and 75% for IgM, with specificities of 100% for both IgG and IgM.

### Statistical Analysis

All statistical analyses were performed using GraphPad Prism, version 8 (GraphPad**^®^** Software). Significant differences in each group were analyzed *via* the Wilcoxon matched-pairs signed-rank test. Statistical significance was set at a p value of less than 0.05. In all instances, n refers to the number of patients analyzed.

## Results

### Establishing the Rules for the CoVITEST Matrix

Blood from SARS-CoV-2-naïve pre-vaccine donors was initially used to establish the four-points matrix of the CoVITEST (see material and method section 2.3). The blood was stimulated and analyzed as described in **Figures 1** and **2**. The 15 or more CD4+IFN-γ+CD69+ events threshold from 40,000 CD4+ T cells was calculated using the average of CD4+IFNg+CD69+ events (5.2) plus 2 standard deviations (2*4.7). Similarly, the ≥5000 IFN-γ MFI from CD4+IFN-γ+CD69+ events threshold was established by the IFN-γ MFI average (2846), plus 2 standard deviations (2*1197). Also, to eliminate any underlying activation unrelated to CoVITEST, there needed to be at least a two-fold increase of CD4+IFN-γ+CD69+ events in the peptide pool sample relative to the negative control (vehicle). Finally, as a blood quality and test indicator, a ≥100 CD4+IFNg+CD69+ events threshold in the positive control (SEB) was established. All four criteria had to be met to determine a patient or donor as having cellular immunity against SARS-CoV-2.

### Assessment of the SARS-CoV-2-Specific T Cell Response Directly From Fresh Whole Blood (CoVITEST) Results Comparable to Those Obtained With Gold Standard Assays (IFN-γELISpot)

**Figure 3:**
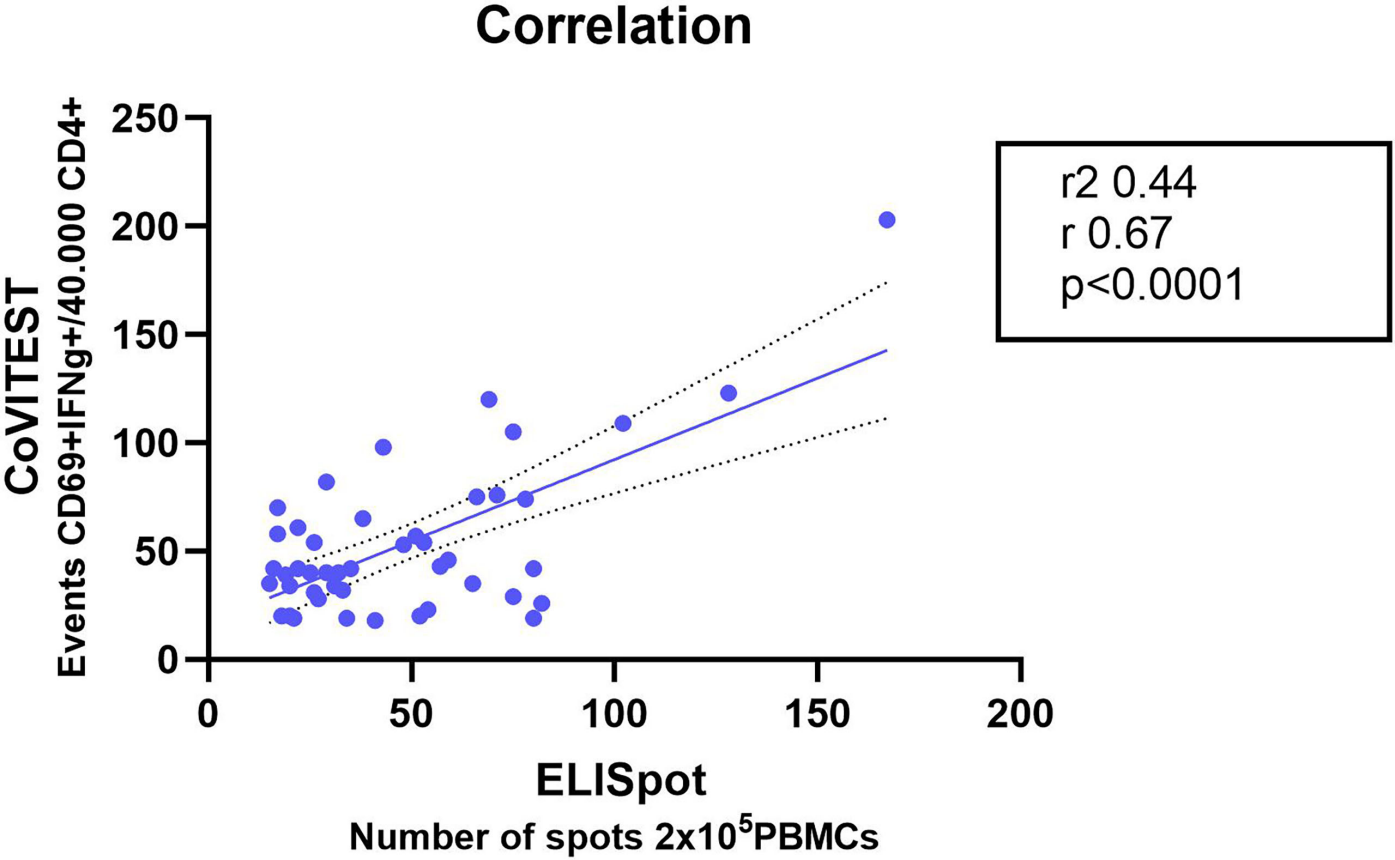
Correlation of SARS-CoV-2-specific T cell response from fresh whole blood CoVITEST with classic T cell IFN-γELISpot Convalescent COVID-19 donors at two weeks, three and six months after the first positive RT-PCR test. Linear regression analysis by comparing the number of CD4+ IFN-γ+ CD69+ T cells from whole blood with specific T cells quantified by IFN-γELISpot (n = 51).

We determined the presence of SARS-CoV-2 specific T cells in 51 mild COVID-19 patients using IFN-γELISpot and CoVITEST. Samples were collected between two weeks and six months after the diagnostic infection of SARS-CoV-2 to define the reliability of CoVITEST for quantification of the SARS-CoV-specific T cell responses. We then compared our results from CoVITEST with those from IFN-γELISpot. We found a good correlation (r = 0.67 p<0.0001) for CoVITEST in whole blood (**Figure 3**).

These results indicated that CoVITEST, which utilizes freshly collected whole blood, is a feasible method for reliable quantification of SARS-CoV-2-specific T cell responses, producing results that are comparable to those obtained with the well-established assays used to analyze T cell responses.

### Distribution of the Cellular and Humoral Immune Response in COVID-19 Patients

**Figure 4:**
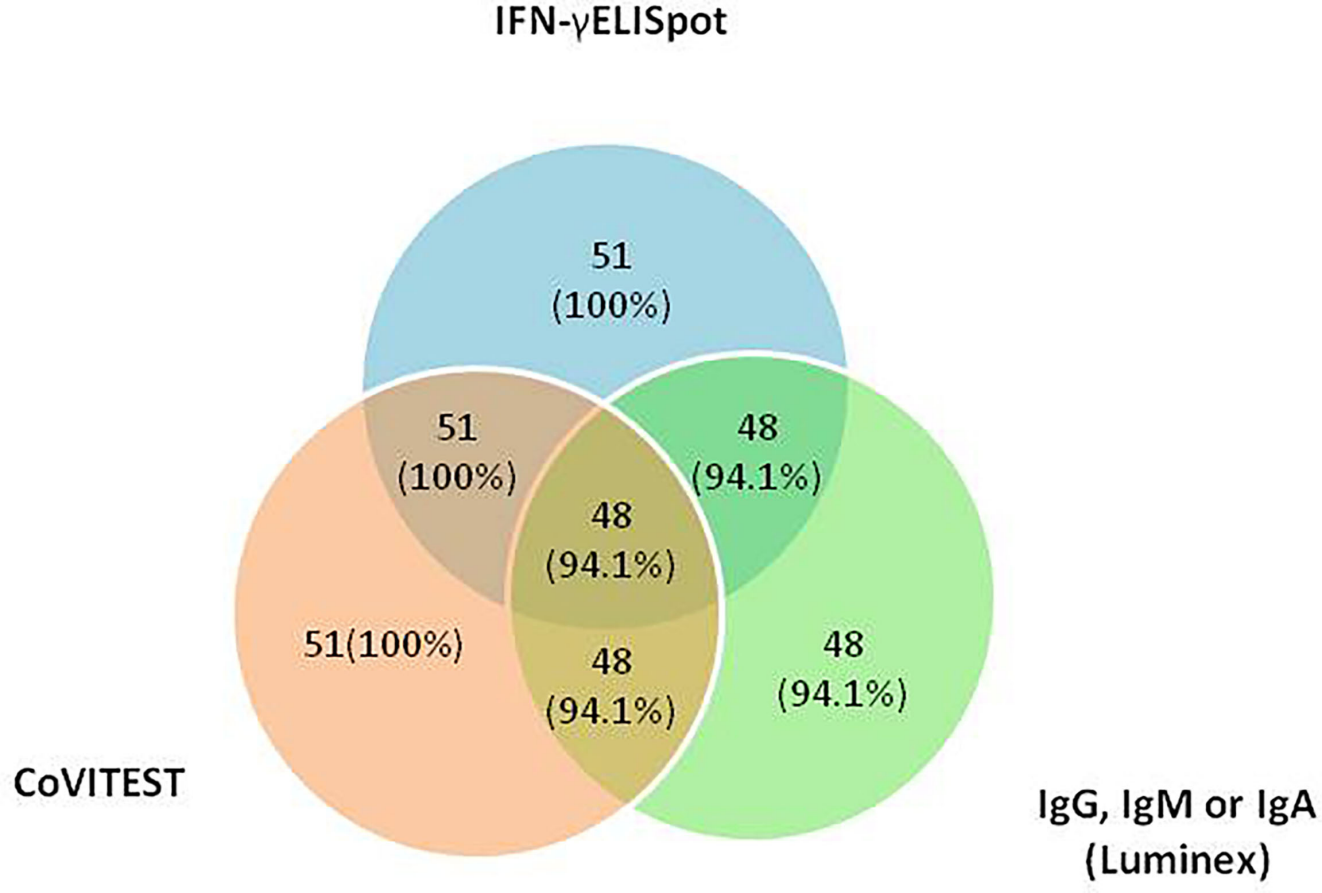
Distribution of the cellular immune response to SARS-CoV-2 by IFN-γELISpot, CoVITEST and antibodies against the Receptor Binding Domain (RBD) of the spike glycoprotein of SARS-CoV-2 (IgG, IgA or IgM) by Luminex in 51 COVID-19 patients.

We analyzed the distribution of the cellular and humoral response in 51 COVID-19 patients, 94.1% had cellular and humoral immune response to SARS-CoV-2 detected by all three methods: IFN-γELISpot, CoVITEST and antibody detection by Luminex (IgG, IgA or IgM); 100% had a cellular immune response to SARS-CoV-2 by IFN-γELISpot and CoVITEST (sensitivity 100%) and, finally, 94.1% had cellular and a humoral immune response to SARS-CoV-2 by CoVITEST and Luminex (**Figure 4**).

Furthermore, we analyzed SARS-CoV-2-specific CD8+ T cell response in COVID-19 patients (n=51) with CoVITEST, 41% developed CD8+ responses (**Supplementary Material, Figure S1**).

### Suitability of the CoVITEST to Evaluate Cellular Immunization After SARS-CoV-2 Vaccination

**Figure 5:**
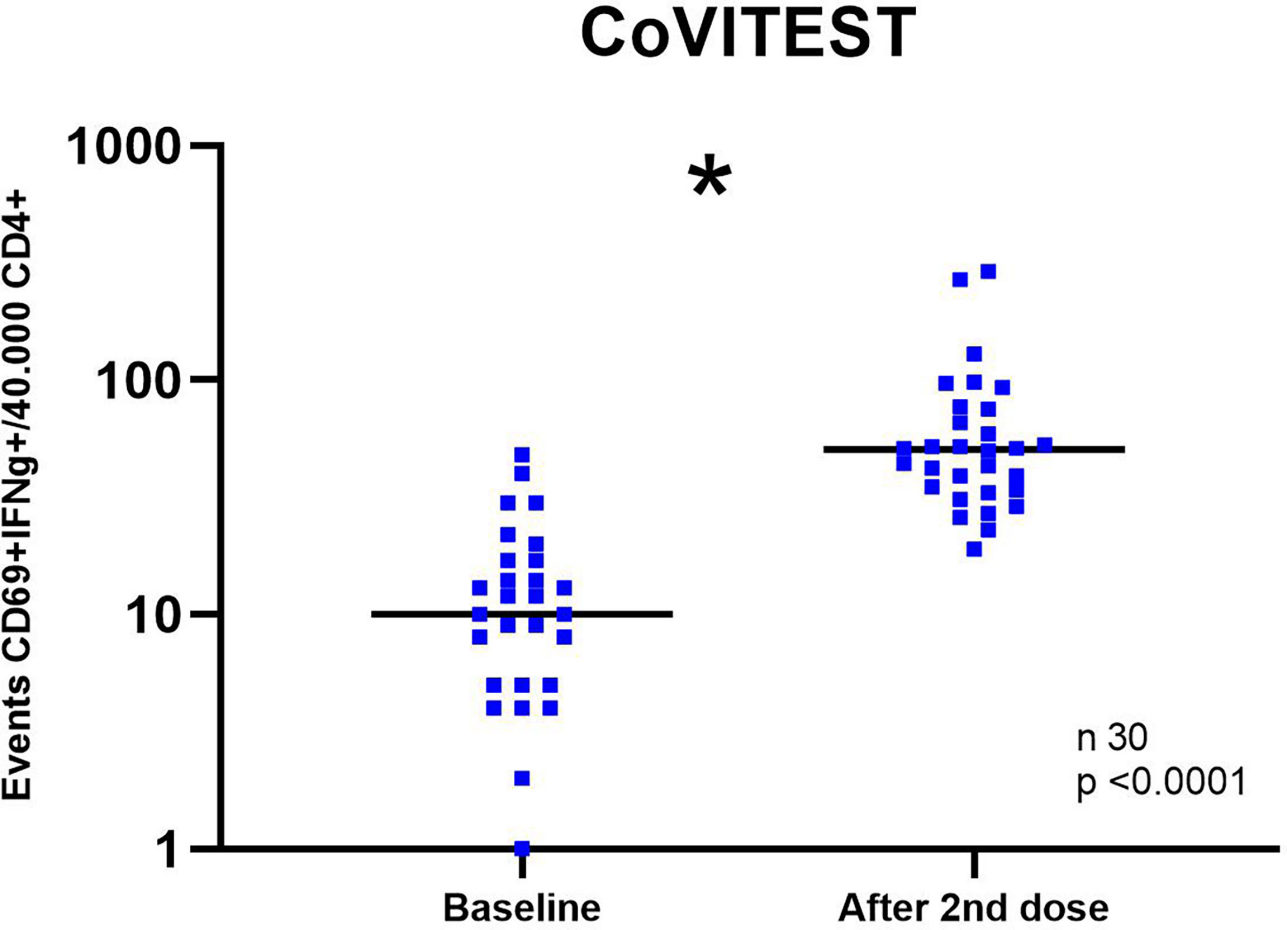
SARS-CoV-2-specific T cell response at baseline and two weeks after the second vaccine dose in healthy unexposed donors. SARS-CoV-2 specific T cells (CD4+ IFN-γ+ CD69+) after stimulation with spike and nucleocapsid SARS-CoV-2 peptide pools at baseline and two weeks after the second vaccine dose. Each dot represents an individual subject. *Statistical comparison at baseline and post-vaccination was performed with the Wilcoxon test.

We characterized the initial kinetics of specific T cells induced by two doses of the mRNA vaccine BNT162b2, mRNA-1273 or ChAdOx1 nCoV-19 (University Oxford/AstraZeneca) in 30 healthy SARS-CoV-2-naïve individuals at baseline (pre-vaccine) and two weeks after the second dose of vaccine. With the CoVITEST method, we were able to determine that 100% of the assessed individuals had raised a SARS-CoV-2–specific T cell response two weeks after the second vaccine dose (**Figure 5**).

## Discussion

Developing worldwide T cell protection against SARS-CoV-2 virus represents a key factor to resolve the COVID-19 pandemic. In this regard, it is known that individuals without any underlying chronic disease can develop protective cellular immunity against SARS-CoV-2 without antibody seroconversion. This baseline cellular response has often been associated with complete recovery from asymptomatic or mild forms of the disease (11, 12). Furthermore, patients unable to produce antibodies (because of inherited or treatment-mediated B cell deficiencies) have also been reported to recover from COVID-19 (14–16). This might be explained, in part, in accordance with what happens in patients with hematological malignancies, in whom CD8+ T cells seem to compensate the lack of humoral immunity and contribute toward improving outcomes of COVID-19 patients. These facts strongly support the role of T cells in the protection against SARS-CoV-2 infection (16) and other SARS (17).

In the same sense, Cucchiari *et al.*, studied cellular and humoral responses in 117 SARS-CoV-2-naïve kidney transplant recipients after receiving the mRNA-1273 vaccine (Moderna) (2). After two vaccine doses, 35 (29.9%) patients developed either IgG or IgM and 64 (54.7%) patients developed cellular responses to the coronavirus (2). Additionally, 50% of the 82 patients with baseline double negativity anti-SARS-CoV-2 IgG and IgM, eventually, *via* infection, developed T cell immunity against the virus (2). Therefore, the status of the immune response against SARS-CoV-2, either by protection triggered by natural infection or after vaccination, especially in immunosuppressed patients, must be evaluated by the study of both reactive T cells and antibody levels.

Although T cell responses are detected in almost all patients who recover from COVID-19 (18), not all COVID-19 and SARS-CoV-2 vaccinated patients have detectable CD8+ specific T cells (11, 12, 19). In this regard, two studies reported that 100% and 89% of convalescent COVID-19 patients had CD4 T cell reactivity, while only 70% and 69% developed CD8+ responses, respectively (12, 19). Because CoVITEST can differentiate between CD4+ and CD8+ T cell responses, our CoVITEST data align with those previously reported, since CD8+ T cell responses are substantially less frequently observed than CD4+ T cells responses (**Figure 4** and **Figure S1**).

Based on our results obtain from patients infected with SARS-CoV-2, we may speculate that CoVITEST could become a reliable and rapid platform to detect reactive T cells against other viruses, including cytomegalovirus, Epstein-Barr virus, and human herpesvirus 6, Mycobacterium spp., and different fungi, such as *aspergillus fumigatus*. This would be especially relevant in patients after allogeneic hematopoietic stem cell and solid organ transplantation, since it is well known that post-transplant therapies include cyclophosphamide and other immunosuppressive agents, which are associated with an increased rate of opportunistic infections (20, 21). Because the immunity of transplant patientss generally assessed by time-consuming and expensive techniques, our CoVITEST platform could be similarly used to rapidly evaluate the immune protection against potentially aggressive pathogens in these fragile patients.

Because T cell responses against SARS-CoV-2 have been demonstrated to play a fundamental role in protecting individuals against severe forms of infection, knowledge of the T cell immune status may contribute toward adjusting therapeutic approaches, particularly in patients with abnormally reduced immune system function.

With this aim, we created CoVITEST, a rapid, simple and accurate in-house method for routine measurement of SARS-CoV-2 reactive T cells from whole blood of selected individuals. As a result, in both SARS-CoV-2 convalescent individuals and healthy-vaccinated donors, CoVITEST was proven to provide accurate results in one working day, comparable to those obtained with the gold standard IFN-γELISpot assays, which analyze cellular immunity but require more time (**Figures 3** and **4**). In that sense, there are other approaches such as surface activation markers (AIM), cytokine release or tetramer-based assays with a good correlation with IFN-γELISpot assays, but they still require a previous PBMC isolation step or have other complexities (long incubation times, prior selection of immunodominant peptides, HLA typing,.), all of which increase the time and cost of the diagnosis (22).

Apart from the detection of specific SARS-CoV-2 T cells, CoVITEST also offers the ability to differentiate CD4+ from CD8+ T cell responses. This is an additional advantage over ELISpot or cytokine release assays such as IGRA, since knowing whether SARS-CoV-2-reactive CD8+ T cells are present is primordial to provide better care to immunocompromised patients (23). In addition, CoVITEST could also be of utility for diagnostic purposes in patients with previous seronegative (or never performed) tests and suspected COVID-19-related conditions (e.g., long-COVID-19 symptoms, post-viral thrombotic complications or interstitial lung diseases, etc.), in whom previous positive tests for SARS-CoV-2 diagnosis were never obtained or performed and could discriminate natural past infection patient form vaccinated individual just split peptides from spike and nucleocapsid in 2 different tubes. As limitation, flow-cytometry requires higher costs in terms of equipment and a higher expertise to analyze data when compare with ELISpot assay.

In conclusion, CoVITEST is an in-house method that provides a rapid measurement of specific SARS-CoV-2 T cells in whole blood in a simple and affordable manner. It can reliably detect spike-specific T cell responses (differentiating CD4+ from CD8+ T cell) induced after SARS-CoV-2 infection or vaccination, and sparing time compared with the standardized IFN-γELISpot assay. Patients with suspected or confirmed immunosuppression may benefit from decision-making protocols regarding re-vaccination against SARS-CoV-2 derived from CoVITEST results. In addition, CoVITEST may be used as a diagnostic tool for SARS-CoV-2 infection in particular clinical situations. Further studies of cellular responses using the CoVITEST assay in patients with different COVID-19 situations are being carried out to replicate and validate this technique as a potential method for routine use.

## Data Availability Statement

The original contributions presented in the study are included in the article/**Supplementary Material**. Further inquiries can be directed to the corresponding authors.

## Ethics Statement

The studies involving human participants were reviewed and approved by Ethical´s Committee of Hospital Clinic of Barcelona (HCB/2020/0967). The patients/participants provided their written informed consent to participate in this study.

## Author Contributions

HC, EG and MJ proposed, directed, discussed, and revised the development of the manuscript. NE, HC, and EG designed the experiments. NE, CG, LH, RM, MF, NM, MT, JB and MV performed the experiments. NE, HC, EG and MJ analyzed and interpreted all the data. NE prepared the figures and wrote the manuscript. MP, JH-R, MM and LM reviewed the manuscript. NE, VO, JH-R, PC and AS recruited the COVID-19 patients and healthy donors, the SARS-Recall patients, and provided all clinical samples and data. All authors contributed to the article and approved the submitted version.

## Funding

The authors declare that this study received funding from CELLNEX TELECOM (5234-20/CPO42837). The funder was not involved in the study design, collection, analysis, interpretation of data, the writing of this article or the decision to submit it for publication.

## Conflict of Interest

The authors declare that the research was conducted in the absence of any commercial or financial relationships that could be construed as a potential conflict of interest.

## Publisher’s Note

All claims expressed in this article are solely those of the authors and do not necessarily represent those of their affiliated organizations, or those of the publisher, the editors and the reviewers. Any product that may be evaluated in this article, or claim that may be made by its manufacturer, is not guaranteed or endorsed by the publisher.
