# Supplementary material for: CoVITEST: A Fast and Reliable Method to Monitor Anti-SARS-CoV-2 Specific T Cells From Whole Blood
Source: Front Immunol. 2022 Jul 5;13:848586. doi: 10.3389/fimmu.2022.848586 (PMC9295597; doi:10.3389/fimmu.2022.848586)
Supplement: Supplementary file 1 [file DataSheet_1.docx]

Supplementary Material

# Supplementary Data

We analyzed SARS-CoV-2-specific CD8+ in COVID-19 patients (n=51) with CoVITEST; 100% of COVID-19 patients had CD4 T cell responses while 41% developed CD8+ responses.

# Supplementary Figure


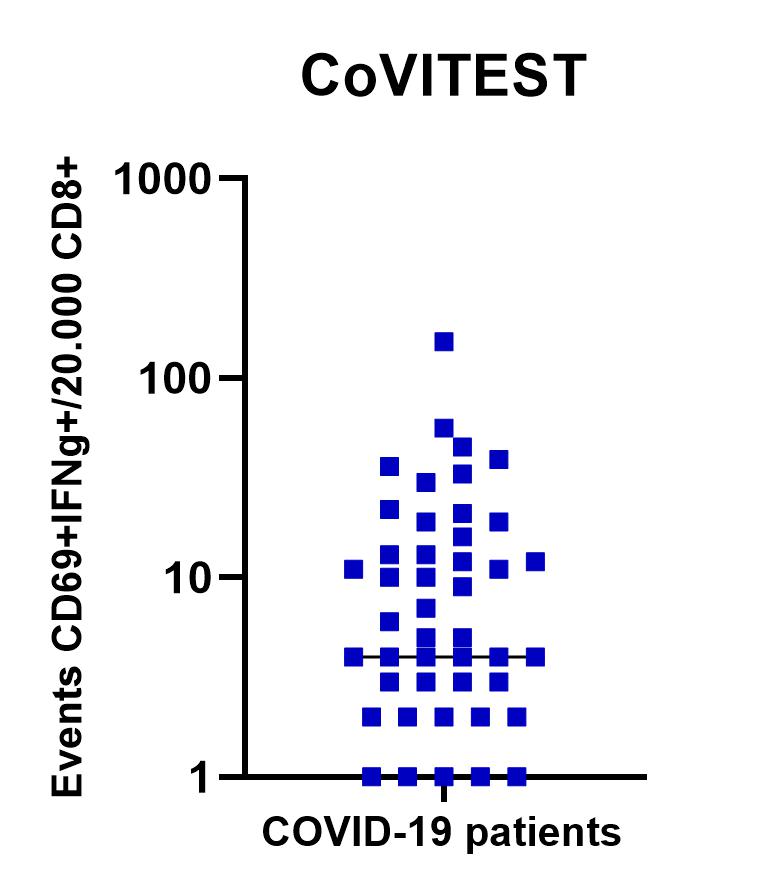


**Supplementary Figure 1.** **Whole blood (**CoVITEST**) quantifies SARS-CoV-2-specific CD8+ in COVID-19 patients.** SARS-CoV-2 specific T cells (CD8+ IFN-γ+ CD69+) after stimulation with spike and nucleocapsid SARS-CoV-2 peptide pools between two weeks and 6 months after the first positive RT-PCR test (n=51). A sample was considered to have reactive T cells when eight or more events were detected in 20,000 CD8+ T cells in the test tube.
